# Effect of various depths of pupation on adult emergence of interspecific hybrid of *Bactrocera carambolae* and *Bactrocera dorsalis*

**DOI:** 10.1038/s41598-022-08295-w

**Published:** 2022-03-10

**Authors:** Agus Susanto, Maura Gita Faradilla, Yayan Sumekar, Dwi Harya Yudistira, Wayan Murdita, Agus Dana Permana, Luciana Djaya, Syifa Nabilah Subakti Putri

**Affiliations:** 1 Department of Plant Pests and Diseases, Faculty of Agriculture, Universitas Padjadjaran, Sumedang, West Java 45363 Indonesia; 2 Pest Forecast Institute, Jatisari, West Java Indonesia; 3 School of Life Sciences and Technology, Institut Teknologi Bandung, Bandung, West Java Indonesia; 4 Bioproduction Science, Graduate School of Agricultural Science, Yamagata University, Yamagata, Japan

**Keywords:** Biological techniques, Genetics, Plant sciences

## Abstract

The depth of the pupation is one of the important factors in the success of fruit flies to become imago. The objective of this study was to evaluate the effect of soil depth on survival, normality dan development time of adult interspecific hybrids of *Bactrocera carambolae* (Drew & Hancock) and *B. dorsalis* (Hendel). The experiments were carried out in a laboratory consisting of seven depths of pupation treatments (4 cm, 10 cm, 20 cm, 30 cm, 40 cm, 50 cm, and 60 cm) with four replications. Soil depth had a significant, negative effect on the survival of the emergence and development time of *B. dorsalis* and *B. carambolae* hybrids. The emergence rate was found to decrease with the increase in pupation depth. The higher survival of the emergence of the hybrid occurred at a depth of 4 cm (95% ± 1.91) and 10 cm (86% ± 2.58), while the lower survival occurred at a depth of 50 cm (12% ± 1.63) and 60 cm (5% ± 3.79). Normal imagoes were found in all soil depths except in 60 cm depth, where all imagoes had abnormal morphology. Means of development time ranged from 8.88 to 10.63 days. The depth of pupation influences the duration of pupae development. The means of development time at a depth of 4–40 cm was similar, but at a depth of 50 cm and 60 cm, a significantly longer time of development were observed. for more effective fruit fly control, this study suggests burying rotten fruit in the soil at a depth of 50 cm or more as a preventive measure for the development of fruit flies.

## Introduction

The carambola fruit fly, *Bactrocera carambolae* and the oriental fruit fly, *Bactrocera dorsalis* are the most destructive pests for various vegetables and fruits^1^. Both species are members of the *B. dorsalis* complex^2^. The *B. carambolae* has a distribution area in a narrower range in Southeast Asia and has recently been introduced to South America^2–6^. Most of the recent studies on *B. carambolae* have been reported frequently in Brazil and other South American countries, because the star fruit fly invasion of this region may lead to serious economic losses^4,7^. *Bactrocera dorsalis* is the main polyphagous fruit fly that attacks more than 300 hosts. The species is widely distributed in over 65 countries in the Asia–Pacific, America, Oceania and Africa, indicating its broad climatic range^2,8–10^. The *B. dorsalis* and *B. carambolae* are considered endemic in the subtropics and tropics area, including Indonesia^11^. Both species attacks on mangoes can reduce their competitiveness in the global market and hinder export activities from Indonesia^12^. The economic impacts caused by fruit fly attacks include a decrease in the quantity and quality of production, an increase in production costs, and consumer refusal^13–15^. Damage caused by *B. dorsalis* and *B. carambolae* in citrus plantation areas in Indonesia has reached 70%^16,17^, while those on papaya plantations even reached 100%^18^.

The *B. carambolae* and *B. dorsalis* may perform interspecific mating due to their genetic closeness and similarity of nucleotide arrangement. As part of the *Bactrocera dorsalis* complex, both species have a close genetic relationship^1^. A previous study shows that interspecific mating in *B. dorsalis* occurs naturally and produces fertile offspring^19^. The mating compatibility between two intently related species to form hybrid species may reach 62%, allowing the hybrid population to develop more prevalent^19–21^. The hybrids are characterized by their wing morphological characters similar to *B. carambolae*, abdomen same as *B. dorsalis*, while the costal wings are in the middle between the two ancestors^20^.

Fruit fly females oviposit their eggs inside the suitable hosts, especially in ripening or ripe fruits and vegetables. After the third instar, the larvae abandon the fruit and burrow into the soil and pupate. When metamorphosis is complete, adult flies burrow upward into the surface, where they stretch and harden their wings before taking off^22^. Because of their unique life history, the development of techniques and control on fruit flies are mostly focused on mature individuals, for example by using methyl eugenol^23,24^, modified traps^25,26^, essence lure^27^, sterile insect technique^28,29^ and botanical pesticides. Meanwhile, efforts to search possibility to control on larva or pupa stages received little attention^30^.

Some of the efforts to control fruit flies at the pupa stage include the application of entomopathogenic nematodes^31,32^, modifying soil moisture^33,34^, and burial of infected fruit^35^. That effort would be more effective if conducted at the right time and methods. Such potential methods can be developed by modifying environmental to be unfavorable conditions for fruit flies during their pupation or burying the infected fruit. The depth of the pupation can interfere with the development of fruit fly pupae and reduce their survival. Therefore information regarding proper depth for burying the infected fruit is pivotal for managing soil conditions. However, studies on interspecific hybrids of *B. carambolae* and *B. dorsalis*, especially pupa survival rates and their time of development in the soil have not been carried out. This study aims to evaluate the effect of soil depth on survival, normality dan development time of adult interspecific hybrids of *Bactrocera carambolae* and *B. dorsalis*.

## Materials and methods

### Insect rearing

A total of 2000 pupae of *B. carambolae* and *B. dorsalis* were obtained from the Center for Plant Pest Forecasting Organisms, Jatisari, West Java. Those pupae were transferred and reared until imagoes in the Pest Laboratory of the Faculty of Agriculture, Universitas Padjadjaran, Indonesia. The rearing method was conducted following the guideline for rearing *Bactrocera* spp. in Laboratorium^36^. The adult fruit flies were kept in a mesh-covered cage sized 50 cm × 50 cm × 50 cm and maintained at room temperature (25–28 °C) with a 12:12 (light:dark) photoperiod. Adults of each species were kept in separated cages. The adults were fed sugar cubes, autolyzed yeast (AY-65), and water. At the age of 3–4 days after imago emergence, males and females of each species were separated to prevent interbreeding. After 2 weeks of emergence, adult fruit flies were considered sexually mature^37,38^, the males of *B. carambolae* were put in a cage together with the *B. dorsalis* females. In this study, we exclude the hybrid from the male *B. dorsalis* and female *B. carambolae*, because those had low survival^39^.

The offspring eggs (F1 hybrids) were harvested using an egging device made of plastic cups with oviposition holes (diameter ± 0.1 mm) on the top side. The egging device was filled with Saipan Mango (*Mangifera odorata*) fruit juice and covered with a plastic wrap on top of it. The adult female laid their eggs into the oviposit hole and the eggs were collected after 2 h. The eggs were transferred to tissue paper and placed in a plastic bowl filled with feed. The larvae were fed made of carrot puree (300 g), mixed with yeast (15 g), nipagin (1.5 g), distilled water (300 ml), and propionic acid (4 ml). The plastic bowl was then put in a container box containing sawdust as the pupation medium. The F1 hybrid pupae were then collected and used for further experiment.

### Pupation medium

The soil for experiment media was acquired from Mango Garden, Tomo Sub District, Sumedang Regency, West Java. Soil samples were collected from five points with an auger at a depth of 0–20 cm. The soil was composed and put all together into a sack. In the laboratory, the soil was sieved through a 1.5 mm mess and sterilized using an autoclave (1 atm) for 15 min at 120 °C. Soil texture was analyzed as sand (7.52%), clay (56.54%), and silt (35.94%). The water capacity of the soil was 45.27%, the porosity was 57.74%, and the permeability was 0.29 cm/h.

### Effect of various depths of pupation on adult emergence

The F1 hybrid pupae were collected after 2 days of formation. Seven various depths were tested for their ability on adult emergence. The treatment consisted of depths of 4, 10, 20, 30, 40, 50, and 60 cm with four replicates. Experiments were carried out using mica plastic, which was formed in a tube with a diameter of 4.5 cm with a height of 30, 60, and 70 cm. The mica tube is filled with soil to a height of 1 cm, then 25 pupae were put on the surface. The tube was then filled with soil to a certain depth according to each treatment. The top of the tube was covered with mesh to prevent adult fruit flies from escaping. After 7 days, the pupae’s conditions in each tube were checked daily until 14 days after the treatment. The number of emerged adults and their morphology were recorded. Adult flies were sorted based on their perfection of abdomen and wings. Adults with imperfect or deformed wings are categorized as abnormal imagoes.

### Data analysis

The number of normal, abnormal and dead adults of *B. dorsalis* and *B. carambolae* hybrid from each depth level was transformed into a percentage. The normality test of the data was carried out before the analysis of the difference in means. Data of the adult survival and length development time were normally distributed, hence statistical analysis is appropriate to do with parametric analysis using univariate analysis of variance. Furthermore, the post hoc test between means was carried out with the Least Significant Different test. The data of adult morphological abnormality were not normally distributed, because there was zero value in treatment for all replicates, so the data analysis was carried out using the non-parametric Kruskal–Wallis test. The post hoc test between means was carried out using the Mann–Whitney test. Statistical tests were performed using Excel and SPSS^®^ version 20 (SPSS Inc. Chicago, IL, USA) and the means were considered different at *P* < 0.05.

## Results and discussion

### Effect of various depth on interspecific hybrid *Bactrocera dorsalis* ♀ and *Bactrocera carambolae* ♂ adult emergence

**Figure 1:**
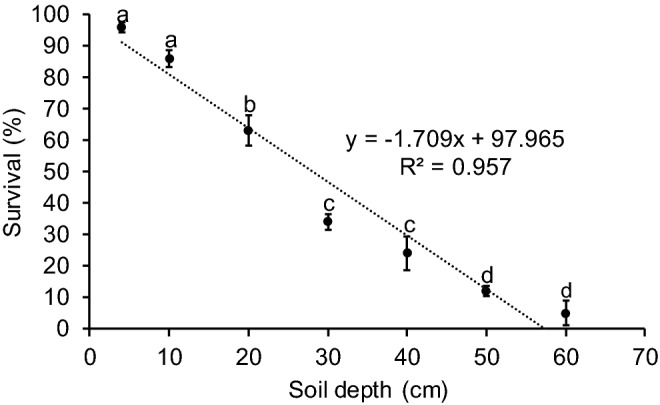
The effect of soil depth on adults of *B. dorsalis* and *B. carambolae* hybrid emergence survival. Different alphabet above the error bar indicate significant differences among the mean of survival.

**Figure 2:**
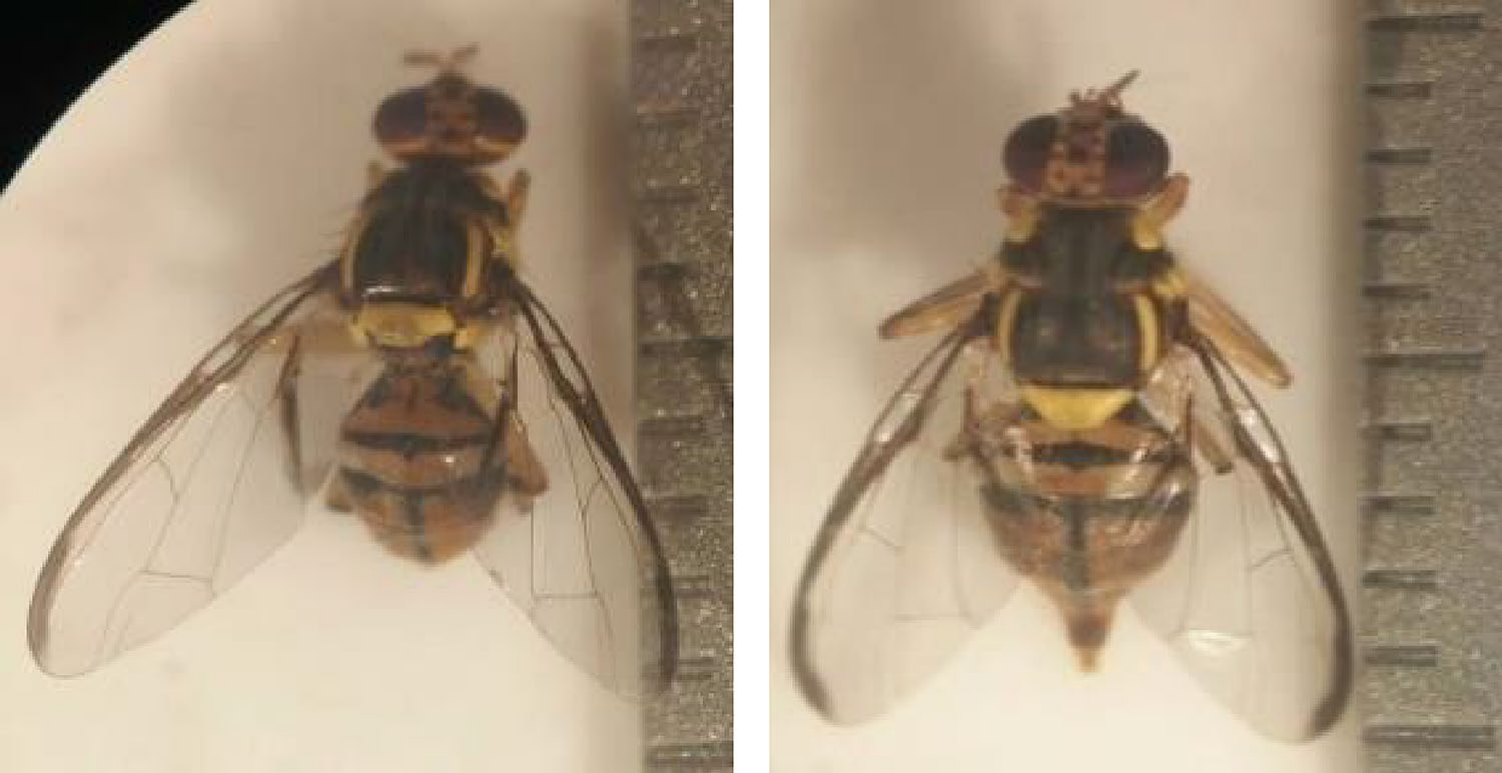
Normal male (left) and female (right) imago of interspecific hybrids morphology*.*

Soil depth had a significant, negative effect on the survival of the emergence of *B. dorsalis* and *B. carambolae* hybrids (R^2^ = 0.979; *P* < 0.001) (Fig. 1). Means of pupa survival rate were significantly different (F = 105.6; *P* < 0.001) between soil depths. The higher survival of the hybrid emergence occurred at a depth of 4 cm (95% ± 1.91) and 10 cm (86% ± 2.58), while the lower survival occurred at a depth of 50 cm (12% ± 1.63) and 60 cm (5% ± 3.79) (Fig. 1). The imagoes those emerged from a depth of 60 cm demonstrated an abnormal morphology with the wrinkle wings (Fig. 2, Table 1). The results of the regression analysis formed the equation y = − 1.709x + 97.965. The prediction result indicated that the effective depth to suppress the emergence of adult *B. dorsalis* and *B. carambolae* hybrids may occur at 57.32 cm depth.

**Table 1:** Effect of soil depth on imago of interspecific hybrid *B. carambolae* ♀ and *B. dorsalis* ♂ abnormality.

Normality category	Depth (cm)						
	4	10	20	30	40	50	60
Normal imago (%)	91 ± 2.52a	83 ± 1.91a	56 ± 6.73bh	24 ± 2.83c	14 ± 1.15d	5 ± 1eg	0 ± 0f
Abnormal imago (%)	5 ± 1eg	3 ± 1ef	7 ± 3deg	10 ± 1.15dg	10 ± 4.76deg	7 ± 1.91deg	5 ± 3.79defg
Dead imago (%)	4 ± 1.63efg	14 ± 2.58dg	37 ± 4.73bc	66 ± 2.58bh	76 ± 5.42ah	88 ± 1.63a	95 ± 3.79a

Values are shown as mean (standard error). Different letters on the same line indicate significant differences at the level of *P* < 0.05 Mann Whitney tests.

Tephritid fruit flies generally pupate below the ground surface. A previous study found that the survival rate of *B. dorsalis* pupae buried at a depth of 0–2 cm is more than 81%, where the soil moisture range from 30 to 70%^40^. Another study reported that the soil depth for *B. carambolae* pupation varied from 2.5 to 5 cm in three different types of soils (sandy, sandy clay loam, and clay loam)^41^. Similar results also occurred in the larvae of the wild olive fruit fly, *B. oleae* (Gmel.). Optimal pupation of the wild olive fruit fly occurs at a depth of less than 5 cm. Most of the *B. oleae* larvae pupate at 3 cm depth and the mean depth of all units is 1.16 cm. The mean of optimum depth differs significantly depending on soil type, humidity, temperature-soil type interaction and soil type-humidity interaction^42^. Pupation on the melon fruit fly, *B. cucurbitae* occurs in the soil at 0.5–15 cm below the soil surface^43^. In this study, the average rate of emergence of hybrid imago began to decrease from a depth of 20 cm and continued to decline to a depth of 60 cm. The decrease in the rate of imago emergence may be due to the difficulty of imago burrowing to the surface as the depth of the soil increases. This is related to the obstacles due to the soil physical and chemical factors which may lead to adult abnormality. The rate of emergence of hybrid imago below 50 cm was relatively low, because of high imago mortality. This result is similar to that of *B. cucurbitae* which is unable to emerge at a depth of 46 cm. The difficulty of the imago to emerge to the surface of the media at a depth of 50 and 60 cm was caused by the insufficient energy possessed by the imago to reach the surface. In another study, it was reported that adult peach flies (*B. zonata*) could emerge from pupae buried in sandy soil at a depth of 40 cm, whereas at 50 cm, adults failed to emerge^30^.

### Effect of pupation depth on imago of interspecific hybrid *B. dorsalis* and *B. carambolae* morphology

The morphology of just emerged imago is characterized by a pale yellowish color, wrinkled wings, a blurred abdomen stripe pattern, and a longer body before maturing into a normal imago. Normal imago showed a clear pattern of wing venation and black stripes on the abdomen, which are the determining feature of fruit fly species (Fig. 2). Disruption of the pupae at the experiment tube can lead to imago abnormality.

This study demonstrated that normal imagoes were found in all soil depths except in 60 cm. At that depth, few imagoes had survived and were all abnormal. Abnormal imagoes were also found in all other soil depths. The percentage of abnormal imago ranged from 3 to 10%. The high imago abnormality was found at the depth of 30 cm (10 ± 1.15 days) and 40 cm (10 ± 4.76 days), while the lowest was found at 10 cm depth of (3 ± 1 cm) (Table 1). Non-parametric K-independent analysis samples showed that means of percentage of all morphological categories were significantly different among all treatments (χ^2^ = 76.2; *P* < 0.001). However, when partial analysis for merely abnormal imagoes was tested, results indicated that there were no differences among the means of percentage of abnormal imagoes among the soil depth (χ^2^ = 8.52; *P* = 0.202).

**Figure 3:**
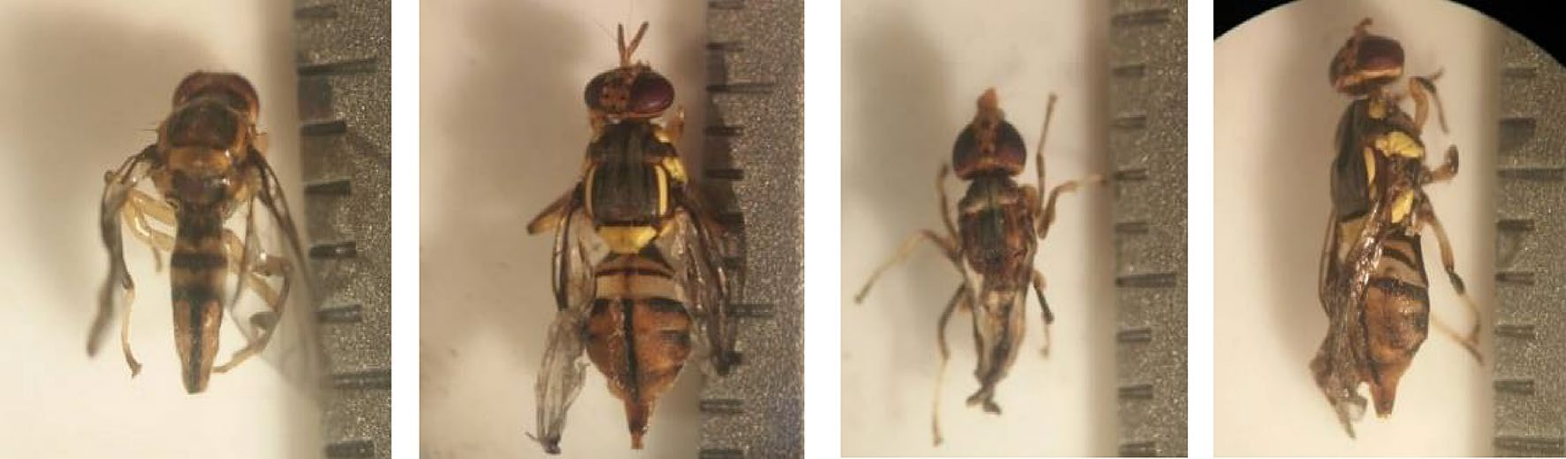
Abnormal imago caused by the pupation depth (30–60 cm).

In general, the morphological abnormality of adult fruit flies occurs in the shape of their wings. The wing development was imperfect, showing a shrunken or wrinkled shape (Fig. 3). Soil physical properties such as pore space, soil particle size, soil compaction, organic matter content and soil moisture may affect the imago success to burrow upward the surface. The larvae prefer to pupate in the soil with a larger soil particle size, making it easier for new imago to emerge from the pupae^42^. Porosity and high organic matter content can reduce soil density that is favorable for imago to emerge and reach the surface^40^. In dense soils, the imago's impose to reach the surface is hampered by the characteristic of soil due to the low total pore space. Therefore, the imago in deeper soil requires more time to reach the surface than that in shallow soil. In this study, the soil porosity was classified as good, so all imagoes have a chance to reach the surface. However, the condition of some of the imago growing abnormally indicated that the depth of the soil had exceeded the imago's ability to develop normally. The other reported that soil texture, porosity, density, temperature and humidity affected the death of pupae and malformed flies^30^. It has been previously reported that soil texture and moisture significantly affect pupa survival of *B. tryoni*^44^, as well as the mature emergence of *Anastrepha ludens* and *A. oblique*^45^.

Finally, humidity may affect the disability of fruit fly imagoes to reach the surface. The *B. dorsalis* larvae showed a strong preference toward pupating in shaded rather than brightly lit areas, as well as in moist rather than dry soil. The ideal moisture levels for the development of *B. dorsalis* ranged from 10 to 60%, at this level emergence rates exceeded 90%^40^. The survival rate of pupae at 70% moisture level was low, and the pupae were unable to survive at soil moistures of 80% or more. Research on other species shows that soil type and soil water content levels are pivotal on pupal mortality of the peach fruit fly (*B. zonata*). Abnormal fruit flies are often found in wet soil conditions^44,45^. However, in another study, pupa depth increased when soil or sand moisture levels increased (*Bactrocera* spp. and Mediterranean fruit flies)^42,46^. In extremely low or high humidity soils, larvae prefer to pupate at shallower depths^44,47^. Low soil moisture negatively affects the vitality and activity of the pupae. For example, due to water evaporation, soils with low water content cause a hard surface and the larvae of *Bractrocera* can only burrow to relatively shallow depths, whereas high moisture content can exacerbate oxygen deficits in deep soils^42^. In the natural habitat, pupation in shallow soil layers due to extreme humidity leads the larvae and pupae more susceptible to predation and less protected from drying and freezing, which can further decrease larval and pupal survival^48,49^.

Based on our result, this study suggests burying rotten fruit in the soil at a depth of 50 cm or more as a preventive measure for the development of fruit flies. Besides, this finding may also be useful to develop the effectiveness of entomopathogenic fungi test related to the depth of the interspecific hybrid *B. carambolae* and *B. dorsalis*. Some soil entomopathogenic fungi isolate such as *Metharizium anisopliae* cause the high mortality of *B. carambolae*^49^. Therefore, the results of this study also have the potential to be developed to improve the performance of entomopathogenic fungi as biological control of fruit flies at a more effective depth level.

### Emergence duration of the interspecific hybrid imago *Bactrocera carambolae* ♀ and *Bactrocera dorsalis* ♂

Means of hybrid development time in the soil ranged from 8.88 to 10.63 days. Treatment with soil depths had a significant effect on the imago development time (F = 8.05, P < 0.001). The depth of pupation influences the duration of pupa development. The results of the post hoc test showed that the means of development time at a depth of 4–40 cm did not show the level of difference, but at a depth of 50 cm and 60 cm, there was a significant difference in development time (Table 2). The delay in the development of the adult fruit fly may be due to the thickness of the soil layer and other soil physical properties. Imago fruit flies buried in the soil with a depth of 30 cm or more, experienced a longer time to reach the soil surface, compared to those in shallow soil. They require more energy to move upwards the surface. With the length of time, they spend inside the soil, their energy requirements may be under-compensated by the unavailability of sufficient oxygen. Furthermore, after being on the surface, fruit flies require sufficient time to stretch their wings to develop perfect morphology. The time required for imagoes to have a normal body and wing is about 25–35 min^50^. The imagoes from the depth of 50 cm and 60 cm experienced longer times in the soil might forfeit their best time to spread their wings. In this study, the experimental tube was kept dry, so the soil moisturized decrease over time. These results are consistent with previous studies which reported that most of the *B. dorsalis* larvae preferred to pupate in less than 4 cm of the soils, while relatively few larvae burrow more than 4 cm when the soils received too little water or too much water^40^.

**Table 2:** The number of interspecific hybrid imago *B. carambolae* ♀ and *B. dorsalis* ♂ appeared on different days.

Treatment in Soil depth (cm)	Development Time (Days after treatment)
4	8.99 ± 0.10^a^
10	8.90 ± 0.06^a^
20	8.88 ± 0.15^a^
30	9.13 ± 0.23^a^
40	9.18 ± 0.33^a^
50	10.17 ± 0.23^b^
60	10.63 ± 0.27^c^

Different letters on the same line indicate significant differences at the level of *P* < 0.05.
